# Small-molecule FTO inhibitor MO-I-500 protects C8-B4 microglial cells from erastin-induced ferroptosis

**DOI:** 10.1038/s41598-026-47881-0

**Published:** 2026-04-18

**Authors:** Denise Greco, Debanjan Das, Jiří Novotný, Mark J. Olsen, Petr Telenský

**Affiliations:** 1 Department of Physiology, Faculty of Science, Charles University, Viničná 1594/7, Prague, 12800 Czech Republic; 2 Department of Pharmacology, First Faculty of Medicine, Charles University and General University Hospital, Prague, Czech Republic; 3 Department of Pharmaceutical Sciences, Midwestern University, Campus Glendale, Glendale, AZ USA; 4 Pharmacometrics Center of Excellence, Midwestern University, Downers Grove, IL USA; 5 Dementia Research Group, International Clinical Research Center of St. Anne ‘s University Hospital Brno, Brno, Czech Republic

**Keywords:** Cell biology, Diseases, Drug discovery, Neurology, Neuroscience

## Abstract

Ferroptosis is a regulated form of cell death driven by iron-dependent lipid peroxidation and the accumulation of lipid-derived reactive oxygen species (ROS). This process has been implicated in various neurological disorders, highlighting the importance of iron metabolism in brain homeostasis. Emerging evidence suggests that epitranscriptomic regulation, particularly N6-methyladenosine (m6A) RNA methylation, may influence ferroptotic signaling pathways. The fat mass and obesity-associated protein (FTO), an m6A RNA demethylase, has been implicated in cellular stress responses; however, its role in ferroptosis remains unclear. Here, we show that inhibition of FTO activity attenuates erastin-induced ferroptosis in C8-B4 microglial cells. Treatment with MO-I-500, a well-characterized FTO inhibitor, significantly decreased erastin-induced cell migration, ROS production, and lipid accumulation. Together, these findings demonstrate that FTO-mediated m6A RNA demethylation plays a crucial role in regulating ferroptotic cell death and suggest that targeting FTO may represent a novel therapeutic strategy for ferroptosis-related neurological disorders.

**Supplementary Information:**

The online version contains supplementary material available at 10.1038/s41598-026-47881-0.

## Introduction

Ferroptosis represents a new form of cell death characterized by the accumulation of lipid reactive oxygen species that results from an increase in free intracellular iron, depletion of the redox glutathione/GPx4/system xc − and the oxidation of membrane polyunsaturated fatty acids (PUFAs)^1^. It involves multiple regulatory axes, including ferritinophagy and mitochondrial pathways. Recent reviews have further delineated the core mechanisms and multiple antioxidant systems that modulate ferroptosis, highlighting its complex regulation in diverse cell types and disease contexts^2,3^.

Under physiological conditions, the balance of iron uptake, storage, and secretion guarantees cellular iron homeostasis. However, when iron exceeds the metabolic necessity, it triggers the Fenton reaction, leading to ROS accumulation and lipid peroxidation^4,5^. Normally, lipid peroxidation is prevented by system xc−, an amino acid antiporter localized in the cell membrane that exchanges cystine and glutamate across the cell membrane^6^. The activity of the system xc − is strictly connected to the synthesis of reduced glutathione GSH, an important intracellular antioxidant^7,8^. In the intracellular space, cystine is reduced to cysteine and used as a substrate in the synthesis of GSH, which in turn is oxidized to glutathione disulfide (GSSG) by glutathione peroxidase 4 (GPX4)^9^. GPX4 oxidizes GSH while reducing the cytotoxic lipid hydroperoxides into the corresponding alcohols, thus inhibiting ferroptosis^10,11^.

Over the past decade, ferroptosis has garnered significant attention for its implications in various pathological conditions, including cancer^12^, neurodegenerative diseases^13^ and ischemia-reperfusion injuries^14^. In the central nervous system (CNS), neuronal death via ferroptosis has been associated with the progression of neurodegenerative disorders such as Alzheimer’s disease^15^ Parkinson’s disease^16^ and amyotrophic lateral sclerosis (ALS)^17^. Notably, both neurons and glial cells are susceptible to ferroptosis.

Glial cells, including astrocytes and microglia, play essential roles in maintaining neuronal homeostasis through metabolic support, synaptic regulation, and immune surveillance^18,19^. Astrocytes contribute to synaptic transmission and plasticity by releasing gliotransmitters, buffering extracellular ions, and recycling neurotransmitters such as glutamate^20^. They also support the glymphatic system, facilitating the clearance of neurotoxic waste like amyloid beta and tau proteins during sleep^21,22^. Microglia, the resident immune cells of the CNS, continuously survey the neural environment, prune synapses, and regulate inflammatory responses to maintain neural integrity^23^. However, under chronic stress or pathological conditions, dysregulation of both astrocytes and microglia can promote neuroinflammation, oxidative stress, and synaptic dysfunction, which further disrupts sleep homeostasis and exacerbates neuronal vulnerability^24,25^. Sustained microglial activation can lead to pro-inflammatory signaling, impaired synaptic integrity, and increased susceptibility to neurodegenerative and psychiatric disorders^24,26,27^. Given their pivotal role in CNS homeostasis and pathology, modulation of glial ferroptosis may represent a promising therapeutic strategy for preserving neuronal function and mitigating disease progression^28^.

Although iron accumulation in microglia cells is commonly observed in the brain during neurodegeneration^29^ the molecular mechanisms that control the ferroptosis pathway remain elusive. Recent evidence highlights a significant connection between ferroptosis and N6-methyladenosine (m6A) RNA methylation, the most prevalent post-transcriptional modification in eukaryotic mRNA^30^. The m6A modifications play a crucial role in modulating ferroptosis by affecting the stability and translation of mRNAs involved in iron metabolism and lipid peroxidation^31^. For instance, the m6A methyltransferase METTL14 has been shown to decrease the stability of FTH1 mRNA, thereby enhancing sorafenib-induced ferroptosis in cervical cancer cells^32^. Conversely, high expression of METTL5, another m6A methyltransferase, represses iron accumulation and ferroptosis, promoting immune evasion in gastric cancer^33^. However, further research is needed to elucidate the correlation of m6A modification and ferroptosis in different diseases.

Here, we investigate the role of FTO m6A RNA demethylase in the microglia ferroptosis process. Our data show that MO-I-500, a well-established FTO inhibitor able to cross the blood-brain barrier via an SVCT2 transporter (Zheng et al. 2014), mitigates the ferroptosis changes induced in vitro in the C8-B4 microglia cell line upon erastin treatment. Therefore, this study reveals a connection between m6A modification and ferroptosis through FTO function, suggesting a new therapeutic strategy for the treatment of neurological and neurodegenerative disorders.

## Materials and methods

### Cell culture

The C8-B4 mouse microglial cell line (ATCC CRL-2540™, American Type Culture Collection, Rockville, MD, USA) was used in all experiments. Cells were maintained in Dulbecco’s Modified Eagle’s Medium (DMEM) supplemented with 10% fetal bovine serum (FBS), 100 U/mL penicillin, and 10 µg/mL streptomycin at 37 °C in a humidified atmosphere containing 5% CO₂.

### Cell treatment

**Fig. 1:**
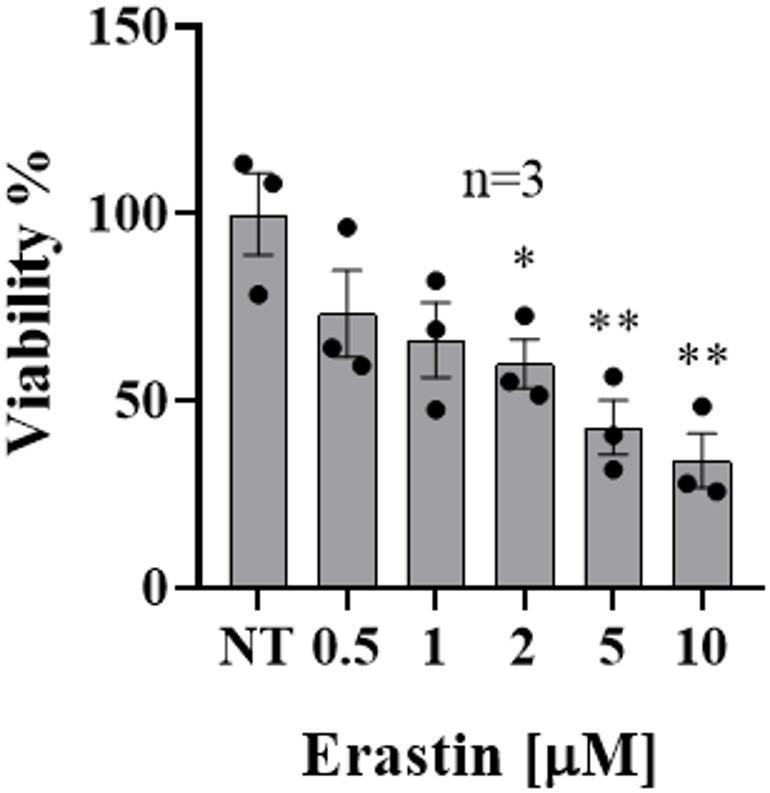
Effects of erastin on cell viability. C8-B4 cells were treated with the indicated concentrations of erastin (0.5, 1, 2, 5, and 10 µM) for 24 h. Cell viability was assessed using the MTT assay. Data is presented as a percentage of control and expressed as mean ± SEM from three independent experiments (*n* = 3). Statistical analysis was performed using one-way ANOVA followed by Dunnett’s post hoc test.

Erastin (MedChemExpress, Cat. No.: HY-15763) was prepared according to the manufacturer’s instructions. Briefly, 0.1828 mL of DMSO was added to each 1 mg vial to obtain a 10 mM stock solution, which was further diluted in DMSO to a final working concentration of 10 µM as determined by MTT assay results (Fig. 1).

MO-I-500 (MW: 317.74 g/mol) was synthesized as previously described^34^, dissolved in DMSO to a 10 mM stock solution, and further diluted to a final working concentration of 1 µM. This therapeutic dose of MO-I-500 was previously determined in neuronal cell line^35^.

For all experiments, C8-B4 cells were treated for 24 h under the following conditions:

1. **Erastin (10 µM)** to induce ferroptosis.
2. **MO-I-500 (1 µM)** to inhibit FTO activity.
3. **Combination treatment** to assess the effect of FTO inhibition on erastin-induced ferroptosis. In the combination group, cells were first pre-treated with erastin for 30 min, followed by the addition of MO-I-500.
4. **Control group**: Cells were treated with 0.1% DMSO vehicle.

All treatments were performed in serum-containing medium at 37 °C in a humidified incubator with 5% CO₂.

### Cell viability assay

Cell viability was assessed using the MTT [3-(4,5-dimethylthiazol-2-yl)-2,5-diphenyl tetrazolium bromide] assay, which quantifies the mitochondrial reduction of MTT to insoluble formazan crystals by metabolically active cells. C8-B4 microglial cells were seeded in 96-well plates at a density of 3 × 10⁴ cells per well and incubated overnight to allow cell adhesion. Cells were then treated with increasing concentrations of erastin (0, 0.5, 1, 2, 5, 10, 20, and 30 µM) for 24 h.

Following treatment, a 12 mM MTT stock solution was prepared in PBS. The culture medium was removed, and 110 µL of fresh medium containing 10% MTT solution was added to each well. Cells were incubated at 37 °C for 2 h, during which metabolically active cells reduced MTT to purple formazan crystals, which were visually confirmed by microscopy. After incubation, all but 25 µL of the MTT-containing medium was carefully removed, and 50 µL of DMSO was added to each well to solubilize the formazan crystals. Plates were gently shaken at room temperature to ensure complete dissolution, followed by an additional 10-minute incubation at 37 °C. Absorbance was measured at 570 nm using a microplate reader (BioTek Synergy HT, Winooski, VA, USA). Cell viability was expressed as a percentage relative to DMSO-treated control cells, which were defined as 100%.

### Scratch assay for cell migration

Cell migration was assessed using a scratch assay. C8-B4 microglial cells were seeded in 6-well plates and cultured at 37 °C until they reached full confluence. A uniform scratch was generated across the cell monolayer using a sterile P1000 pipette tip. Images of each well were acquired immediately after scratching (time 0 h, T0) using an inverted microscope.

Following scratch generation, the culture medium was removed and replaced with serum-free medium containing MO-I-500, erastin, or their combination, as described above. Cells were incubated under these conditions for 24 h to minimize cell proliferation and ensure that changes in scratch area primarily reflected migratory activity. After treatment, cells were gently washed with ice-cold PBS and stained with crystal violet staining solution for 1 min. Excess stain was removed, and wells were rinsed three times with PBS. Representative images were captured at 24 h post-scratch (T24).

Cell migration was quantified by measuring the reduction in scratch area using ImageJ software (v1.53e; Rasband W.S., ImageJ, U.S. National Institutes of Health, Bethesda, MD, USA, https://imagej.net/ij/). Migration was calculated using the following formula:

$$\:\text{Migration }(\%)=\left[\frac{A\left(T0\right)-A\left(T24\right)}{A\left(T0\right)}\right]\times\:100$$

where *A* represents the scratch area at time 0–24 h.

### Mitochondrial superoxide assay

Mitochondrial superoxide production was assessed using the Invitrogen MitoSOX Red Mitochondrial Superoxide Indicator (Thermo Fisher Scientific), a live-cell–permeant fluorogenic dye that selectively accumulates in mitochondria and fluoresces upon oxidation by superoxide.

A MitoSOX Red stock solution was prepared according to the manufacturer’s instructions and diluted to a final working concentration of 5 µg/mL. C8-B4 microglial cells were seeded in 24-well plates at a density of 6 × 10⁴ cells per well and cultured until approximately 70% confluence. Cells were then treated for 24 h with MO-I-500, erastin, or their combination, as described above. Control cells were treated with 0.1% DMSO vehicle.

Following treatment, cells were incubated with MitoSOX Red for 30 min at 37 °C in the dark, washed twice with PBS to remove excess dye, and harvested by trypsinization. Cells were resuspended in HBSS and immediately analysed by flow cytometry using a BD LSR flow cytometer (BD Biosciences, NJ, USA). MitoSOX fluorescence was detected in the PE channel (excitation 510 nm, emission 580 nm). Forward and side scatter parameters were used to exclude debris and cell aggregates. At least 10,000 events per sample were collected.

Data were analysed using FlowJo software (FlowJo_v10.9.0_CL, https://flowjo.com/), and the percentage of MitoSOX-positive cells was quantified relative to total viable cells and normalized to DMSO-treated controls.

### Mitochondrial mass and mitochondrial membrane potential (MMP)

Changes in mitochondrial mass and mitochondrial membrane potential (MMP) were evaluated using MitoTracker Green FM (MTG; M7514) and MitoTracker Red CMXRos (MTR; M7512) (Thermo Fisher Scientific, MA, USA), respectively. Both dyes passively diffuse across the plasma membrane and accumulate in mitochondria; MTG reports mitochondrial content, while MTR is sensitive to MMP.

Stock solutions were prepared according to the manufacturer’s instructions, and cells were stained at a final concentration of 40 nM. C8-B4 microglial cells were seeded in 24-well plates at a density of 6 × 10⁴ cells per well. Upon reaching approximately 70% confluence, cells were treated for 24 h with MO-I-500, erastin, or their combination. Control cells received 0.1% DMSO.

After treatment, cells were washed with PBS, harvested by trypsinization, and incubated with MTG and MTR for 30 min at room temperature in the dark. Cells were washed twice with PBS, resuspended in HBSS, and immediately analyzed using a BD LSR flow cytometer (BD Biosciences, NJ, USA). Forward and side scatter parameters were used to exclude debris and cell aggregates, and at least 10,000 events per sample were collected. Data were analyzed using FlowJo software (FlowJo_v10.9.0_CL, https://flowjo.com/), and mitochondrial mass and MMP were quantified as the percentage of MTG- or MTR-positive cells, expressed as fold change relative to DMSO-treated control cells.

### Lipid accumulation assessment

Intracellular lipid accumulation was assessed using BODIPY 493/503 staining. BODIPY 493/503 (MedChemExpress, Cat. No.: HY-D1614) is a fluorescent probe that selectively labels neutral lipids within lipid droplets. Although this probe does not directly detect lipid peroxidation, alterations in neutral lipid content reflect lipid metabolic remodeling associated with ferroptosis and may influence cellular susceptibility to oxidative lipid damage.

The stock solution was prepared according to the manufacturer’s instructions by dissolving 1 mg of BODIPY 493/503 in DMSO to obtain a 10 mM stock solution. The stock was subsequently diluted in PBS to generate a working concentration of 2 µM.

C8-B4 microglial cells were seeded in 24-well plates at a density of 6 × 10⁴ cells per well. Upon reaching approximately 70% confluence, cells were treated for 24 h with MO-I-500, erastin, or a combination of MO-I-500 and erastin. Control cells were treated with 0.1% DMSO vehicle. After treatment, cells were washed with PBS, harvested by trypsinization, and incubated with BODIPY 493/503 for 15 min at room temperature in the dark. Following staining, cells were washed twice with PBS and resuspended in Hanks’ Balanced Salt Solution (HBSS).

Flow cytometric analysis was performed using a BD LSR flow cytometer (BD Biosciences). Instrument settings were optimized using unstained cells and kept constant across all experimental conditions. At least 10,000 events were acquired per sample. Cells were gated based on forward and side scatter (FSC/SSC) to exclude debris, followed by doublet discrimination using FSC-A versus FSC-H. Vehicle-treated cells served as negative controls to establish gating thresholds. BODIPY 493/503 fluorescence was detected using the FITC channel. Data were acquired using BD FACSDiva software and analysed with FlowJo software (FlowJo_v10.9.0_CL, https://flowjo.com/).

Lipid accumulation was quantified as the percentage of BODIPY-positive cells and expressed as a fold change relative to the control group.

### Intracellular ROS measurement

Intracellular reactive oxygen species (ROS) levels were assessed using 2′,7′-dichlorofluorescein diacetate (DCFDA; Sigma-Aldrich). C8-B4 microglial cells were cultured in 12-well plates and treated for 24 h with MO-I-500, erastin, or their combination, as described above. Following treatment, the culture medium was removed, and cells were incubated with 10 µM DCFDA in PBS for 30 min at 37 °C in the dark. After staining, cells were washed three times with PBS to remove excess dye.

Fluorescence images were acquired using a fluorescence microscope (AIF, Arsenal, Czech Republic) with excitation/emission wavelengths of 495/527 nm. DCFDA fluorescence intensity was quantified using ImageJ software by measuring the mean gray value per image using identical acquisition and analysis settings. ROS levels are expressed as fold change relative to DMSO-treated control cells.

Additionally, ROS levels were assessed by flow cytometry. C8-B4 microglial cells were seeded in 24-well plates at a density of 6 × 10⁴ cells per well. Upon reaching approximately 70% confluence, cells were treated for 24 h with MO-I-500, erastin, or a combination of MO-I-500 and erastin. Control cells were treated with 0.1% DMSO vehicle. After the treatment, C8-B4 cells were incubated with 10 µM DCFDA for 30 min at 37 °C in the dark, washed twice with PBS to remove excess dye, and harvested by trypsinization. Cells were resuspended in HBSS and immediately analysed by flow cytometry using a BD LSR flow cytometer (BD Biosciences, NJ, USA). ROS levels were quantified as the percentage of DCFDA-positive cells relative to DMSO-treated controls, expressed as fold change.

### Protein isolation and western blot analysis

Protein levels were determined by Western blot analysis as previously described in^35^. Cells were washed with ice-cold PBS, scraped, and harvested by centrifugation at 1,000 × g for 10 min at 4 °C. The resulting pellet was resuspended in TMES buffer (20 mM Tris, 3 mM MgCl2, 1 mM EDTA, 250 mM sucrose, pH 7.4) supplemented with protease inhibitors (cOmplete Protease Inhibitor Cocktail, Roche). Protein concentration was determined using the Bicinchoninic Acid (BCA) Protein Assay.

Samples were solubilized in Laemmli buffer, and equal amounts of protein were loaded onto 10% sodium dodecyl sulfate–polyacrylamide gels (SDS-PAGE). After electrophoresis, to verify the equal protein loading and transfer, reversible Ponceau S staining was performed.

Proteins were transferred onto nitrocellulose membranes. Membranes were blocked for 1 h at room temperature with 5% (w/v) non-fat dry milk in Tris-buffered saline (10 mM Tris, 150 mM NaCl, pH 8) containing 0.1% (v/v) Tween 20 (TBS-T buffer).

After blocking, membranes were incubated with primary antibodies overnight at 4 °C. Membranes were then washed three times for 10 min each with wash buffer (TBS with 3% Tween 20), followed by incubation with a horseradish peroxidase-labeled secondary antibody for 1 h at room temperature. After three additional washes, protein bands were visualized using enhanced chemiluminescence according to the manufacturer’s instructions. Immunoblots were scanned and quantitatively analyzed using ImageJ software. The signal from the target proteins was normalized to the β-actin signal (Table 1).

**Table 1:** Western blot antibody.

Antigen	Host	Clonality	Dilution	Manufacturer	Catalogue nr
Ferritin heavy chain	Mouse	Monoclonal	1:1000	Santa Cruz	sc-376,594
GPX4	Mouse	Monoclonal	1:1000	Santa Cruz	sc-166,120
ACSL4	Mouse	Monoclonal	1:1000	Santa Cruz	sc-365,230
Β-actin	Mouse	Monoclonal	1:40000	Santa Cruz	sc-130,065
Mouse IgG HRP-Linked Whole Ab	Sheep	Unknown	1:10000	GE Healthcare UK limited	NXA931V

### Flow cytometry analyses

Flow cytometry–based assays were performed using a BD LSR flow cytometer (BD Biosciences, NJ, USA). For all experiments, forward scatter (FSC) and side scatter (SSC) parameters were used to exclude debris, and doublets were excluded using FSC-A versus FSC-H. Unstained cells and vehicle-treated cells (0.1% DMSO) were used as negative controls to define background fluorescence and gating thresholds. At least 10,000 events per sample were acquired. Data were analyzed using FlowJo software (FlowJo_v10.9.0_CL, https://flowjo.com/), and results were expressed as the percentage of positive cells relative to DMSO-treated controls.

1. Intracellular ROS (DCFDA)

Intracellular ROS were assessed using DCFDA dye. C8-B4 cells were incubated with 10 µM DCFDA for 30 min at 37 °C in the dark, washed twice with PBS to remove excess dye, and harvested by trypsinization. Cells were resuspended in HBSS and immediately analysed by flow cytometry using a BD LSR flow cytometer (BD Biosciences, NJ, USA). ROS levels were quantified as the percentage of DCFDA-positive cells relative to DMSO-treated controls, expressed as fold change. Representative flow cytometry plots illustrating the gating strategy and fluorescence distribution are shown in Supplementary Figure S1.

2. Mitochondrial Superoxide (MitoSOX Red)

Mitochondrial superoxide levels were assessed using MitoSOX Red (Thermo Fisher Scientific). Cells were stained at a final concentration of 5 µg/mL for 30 min at 37 °C in the dark, washed twice, and analyzed in the PE channel (excitation 510 nm, emission 580 nm). Fluorescence was quantified as the percentage of MitoSOX-positive cells relative to controls. Representative flow cytometry plots illustrating the gating strategy and fluorescence distribution are shown in Supplementary Fig. S2.

3. Lipid Accumulation (BODIPY 493/503)

Intracellular neutral lipid accumulation was measured using BODIPY 493/503 (MedChemExpress, Cat. No. HY-D1614), which selectively labels neutral lipids in lipid droplets. A 10 mM stock solution was prepared in DMSO and diluted in PBS to a 2 µM working solution. C8-B4 cells were seeded at 6 × 10⁴ cells per well in 24-well plates and treated for 24 h with MO-I-500, erastin, or their combination. After treatment, cells were washed, harvested, and stained with BODIPY 493/503 for 15 min at room temperature in the dark. Cells were washed twice, resuspended in HBSS, and analyzed by flow cytometry using the FITC channel (excitation 488 nm, emission 510–530 nm). Representative flow cytometry plots illustrating the gating strategy and fluorescence distribution are shown in Supplementary Fig. S3.

4. Mitochondrial Mass and Membrane Potential (MitoTracker)

Mitochondrial mass and membrane potential (MMP) were evaluated using MitoTracker Green FM (MTG; M7514) and MitoTracker Red CMXRos (MTR; M7512). Cells were stained at a final concentration of 40 nM for 30 min at room temperature in the dark, washed, and analyzed by flow cytometry. MTG reports mitochondrial mass, while MTR is sensitive to MMP. Data were quantified as the percentage of MTG- or MTR-positive cells relative to DMSO-treated controls. Representative flow cytometry plots illustrating gating strategy and fluorescence distribution are shown in Supplementary Figure S4.

### Statistical analysis

Statistical analyses were performed using GraphPad Prism software version 8.0 (GraphPad Software, San Diego, CA, USA, https://www.graphpad.com/). Differences between groups were analyzed by two-way ANOVA analysis. Data are expressed as mean ± SEM. The symbols * or # indicate a statistically significant difference in *post-hoc* analysis as follows: */# ~ *p* < 0.05, **/## ~ *p* < 0.01, ***/###~ *p* < 0.001, ****/#### ~ *p* < 0.0001.

## Results

### Erastin reduces C8-B4 cell viability

Cell viability was assessed using the MTT assay. Erastin treatment decreased C8-B4 cell viability in a dose-dependent manner (Fig. 1). One-way ANOVA revealed a significant effect of erastin on cell viability (*p* = 0.0038), with significant reductions observed at 2 µM (*p* < 0.05), 5 µM (*p* < 0.01), and 10 µM (*p* < 0.01) compared to vehicle-treated controls. Cell viability at 10 µM was reduced by approximately 66% relative to the control.

### FTO inhibition reduces the microglia migration induced by erastin treatment

**Fig. 2:**
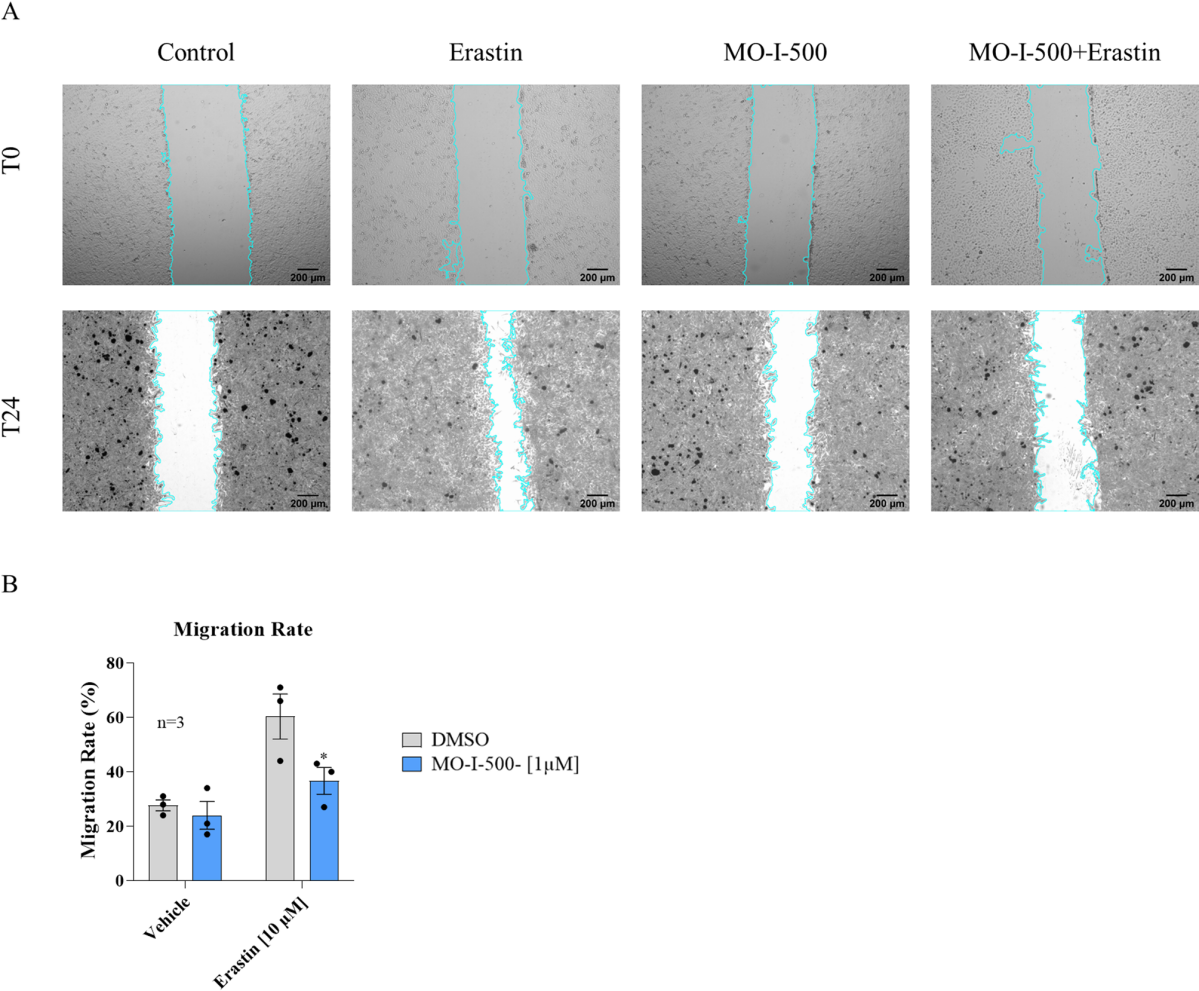
MO-I-500 significantly reduced erastin–induced microglial migration. C8-B4 cells were seeded in 6-well plates and cultured at 37 °C in a 5% CO₂ atmosphere until confluence. A uniform scratch was generated using a sterile pipette tip, and cells were subsequently treated with erastin and/or MO-I-500 for 24 h under serum-free conditions. Representative images were acquired immediately after scratching (T0) and after 24 h (T24) (**A**). Cell migration was quantified by measuring the scratched area using ImageJ software and expressed as migration rate (**B**). Data are presented as mean ± SEM from three independent experiments (*n* = 3). Statistical analysis was performed using two-way ANOVA followed by Sidak’s multiple comparisons test.

To examine the effect of FTO inhibition on erastin-induced changes in microglial motility, the migratory capacity of C8-B4 cells was assessed using a scratch migration assay. Erastin treatment significantly altered cell migration compared to vehicle-treated controls (Fig. 2). Two-way ANOVA revealed a significant main effect of erastin (*p* = 0.0035) and MO-I-500 (*p* = 0.0392). Co-treatment with MO-I-500 significantly reduced erastin-induced migration compared to erastin treatment alone (*p* < 0.05).

### FTO inhibition ameliorates erastin-induced oxidative damage

**Fig. 3:**
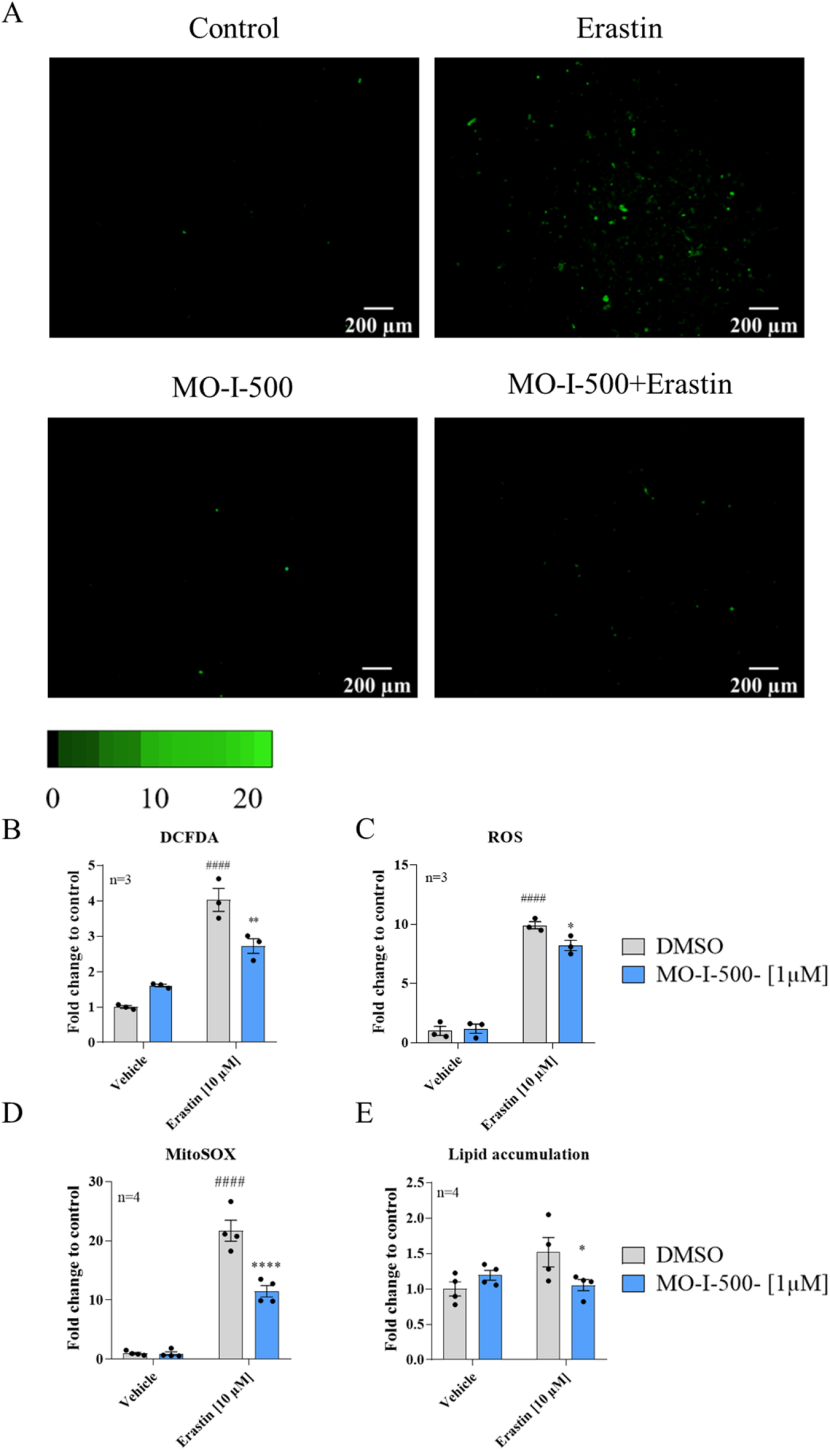
FTO inhibition using MO-I-500 significantly reduced the erastin–induced intracellular ROS and lipid accumulation in C8-B4 microglia cells. Intracellular reactive oxygen species (ROS) levels were assessed using DCFDA staining and visualized by fluorescence microscopy (scale bar = 200 μm) (**A**). Quantification of DCFDA fluorescence intensity is shown in (**B**). Intracellular ROS levels were further assessed by flow cytometry (**C**). Mitochondrial superoxide production was evaluated using MitoSOX Red and analyzed by flow cytometry (**D**). Lipid accumulation was assessed using the BODIPY probe and analyzed by flow cytometry (**E**). C8-B4 cells were treated with erastin and/or MO-I-500 as indicated. Data are presented as mean ± SEM from three or four independent experiments, as indicated in the figures. Statistical analyses were performed using two-way ANOVA followed by Tukey’s or Sidak’s multiple comparisons tests, as indicated. Representative flow cytometry plots illustrating gating strategy and fluorescence distribution are shown in Supplementary Fig. S1-3.

Intracellular reactive oxygen species (ROS) levels were first assessed using DCFDA fluorescence microscopy (Fig. 3A–B). Erastin treatment significantly increased intracellular ROS levels in C8-B4 microglial cells compared to vehicle-treated controls (3-fold increase). Two-way ANOVA followed by Tukey’s post-hoc test revealed a significant main effect of erastin (*p* < 0.0001) and a significant interaction between erastin and MO-I-500 (*p* = 0.0044). Co-treatment with MO-I-500 significantly attenuated erastin-induced ROS levels, resulting in a 32% reduction relative to erastin alone (*p* < 0.05).

Intracellular ROS levels were further evaluated by flow cytometry (Fig. 3C), confirming the microscopy findings. Erastin significantly increased the percentage of ROS-positive cells relative to controls (9-fold increase), while MO-I-500 co-treatment significantly reduced this increase. Two-way ANOVA revealed a significant main effect of erastin (*p* < 0.0001) and a significant interaction between erastin and MO-I-500 (*p* = 0.0397). Co-treatment with MO-I-500 attenuated erastin-induced ROS levels, resulting in a 17% reduction relative to erastin alone (*p* = 0.0276).

Mitochondrial superoxide production was subsequently evaluated using MitoSOX Red and analyzed by flow cytometry (Fig. 3D). The percentage of positive cells was compared across groups, and results were expressed as fold change relative to vehicle-treated controls. Erastin treatment resulted in a significant increase in mitochondrial superoxide levels compared to vehicle-treated controls (20-fold increase, *p* < 0.0001). Two-way ANOVA followed by Tukey’s post-hoc test revealed significant main effects of erastin (*p* < 0.0001), MO-I-500 (*p* = 0.0003), and their interaction (*p* = 0.0003). Co-treatment with MO-I-500 significantly attenuated erastin-induced mitochondrial superoxide accumulation, resulting in a 50% reduction relative to erastin alone (*p* < 0.0001).

Finally, lipid accumulation was assessed using the BODIPY probe (Fig. 3E). The percentage of positive cells was compared across groups, and results were expressed as fold change relative to vehicle-treated controls. Two-way ANOVA followed by Sidak’s multiple comparisons test revealed a significant interaction between erastin and MO-I-500 (*p* = 0.0234), and MO-I-500 co-treatment significantly reduced erastin-induced lipid accumulation, resulting in a 33% reduction relative to erastin alone (*p* < 0.05).

### Effect of erastin treatment on mitochondrial function

**Fig. 4:**
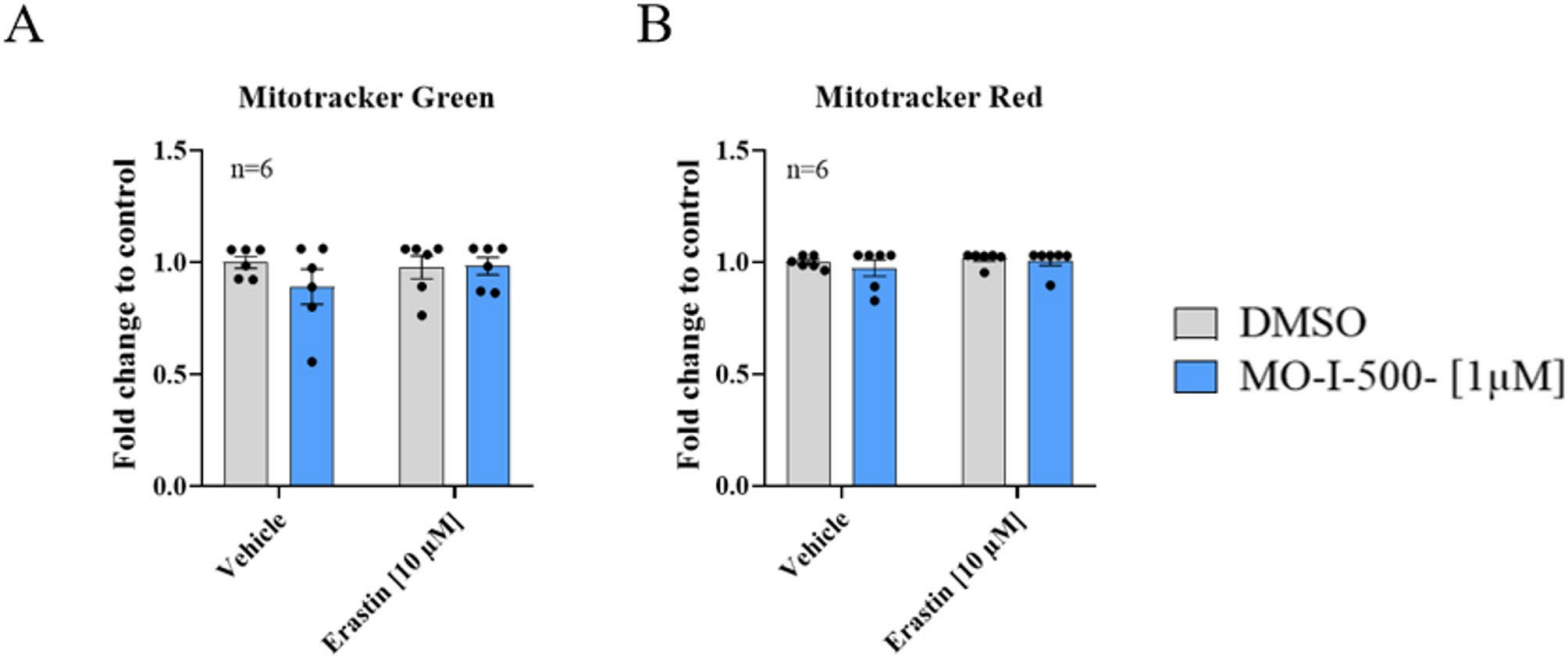
Effects of MO-I-500 on mitochondrial mass and mitochondrial membrane potential in erastin treated C8-B4 cells. Mitochondrial mass and membrane potential were assessed by flow cytometry using MitoTracker Green FM and MitoTracker Red CMXRos, respectively. Data was analyzed using FlowJo software. The percentage of positive cells was compared across groups, and results are expressed as fold change relative to the control group. Values are presented as mean ± SEM from six independent experiments (*n* = 6). Statistical significance was assessed using two-way ANOVA. Representative flow cytometry plots illustrating gating strategy and fluorescence distribution are shown in Supplementary Fig. S4.

To investigate the effect of erastin on mitochondrial function, mitochondrial mass and mitochondrial membrane potential were evaluated via flow cytometry using MitoTracker Green FM and MitoTracker Red CMXRos, respectively (Fig. 4). Analysis of the percentage of positive cells revealed no significant changes in mitochondrial mass or membrane potential following treatment with erastin, MO-I-500, or their combination compared to control cells. Two-way ANOVA followed by Sidak’s multiple comparisons test showed no significant main effects or interactions, indicating that neither erastin treatment nor FTO inhibition compromised mitochondrial biogenesis or mitochondrial membrane potential under the experimental conditions tested.

## Discussion

Ferroptosis is an iron-dependent form of cell death recently linked to neuroinflammation and the progression of neurodegenerative disorders^36^. Recent evidence indicates that ferroptotic stress in microglia can initiate inflammatory signaling cascades that convert astrocytes into neurotoxic states, ultimately promoting non–cell-autonomous neuronal death in amyotrophic lateral sclerosis (ALS) and other neurodegenerative diseases^37^. These findings highlight glial ferroptosis as a potentially important contributor to neurodegenerative pathology.

To address the role of glial ferroptosis, its epitranscriptomic regulation in neurodegeneration, we evaluated the effects of the small molecule FTO inhibitor MO-I-500 on erastin-induced ferroptosis in C8-B4 microglia cells. The efficacy and specificity of MO-I-500 have been demonstrated in neural and glial models, where it reduces oxidative stress, preserves mitochondrial function, and modulates cellular stress responses via inhibition of FTO-mediated m6A demethylation^34,38^. However, genetic approaches, such as FTO knockdown or overexpression, were not performed in this study. Our results show that MO-I-500 attenuated several ferroptosis-associated features induced by erastin, including intracellular ROS accumulation, mitochondrial superoxide production, and lipid accumulation. These findings suggest that FTO inhibition can modulate oxidative and lipid stress responses associated with ferroptosis in microglia. In addition, MO-I-500 reduced erastin-induced microglial motility, indicating that FTO activity may influence microglial functional behaviour under oxidative stress conditions. Importantly, migration is interpreted here as a stress-associated functional response rather than a definitive marker of microglial activation.

Oxidative stress, iron accumulation, and lipid peroxidation are key mechanisms driving ferroptosis^39^. When the intracellular ROS levels exceed the antioxidant defenses, oxidative damage occurs in membrane lipids, causing lipid peroxide accumulation, thereby enhancing ferroptotic cell death^40^. Oxidative stress–driven ferroptosis has been implicated in multiple neurological disorders^41,42^. A recent study demonstrated that NADPH oxidase 4 (NOX4), contributes to astrocyte ferroptosis through oxidative stress-induced lipid peroxidation and mitochondrial dysfunction, identifying NOX4 as a potential target for the treatment of Alzheimer’s disease^43^. In line with this, deficiency of Nrf2 in astrocytes of AD patients and 3×Tg mice induces NOX4 upregulation, which leads to ferroptosis oxidative stress-induced ROS, downregulation of heme oxygenase-1 (HO-1) and glutathione peroxidase 4 (GPX4), upregulation of cystine glutamate, increased lipid peroxidation, DNA oxidation, and mitochondrial fragmentation^44^. Notably, recent evidence further supports the central role of GPX4 and Nrf2 signaling in counteracting ferroptosis in AD. The natural compound Thonningianin A was shown to inhibit ferroptosis by activating the AMPK/Nrf2 pathway, enhancing GPX4 activity, reducing lipid peroxidation, iron accumulation, and mitochondrial dysfunction, and improving neurobehavioral outcomes in experimental AD models^45^. Therefore, targeting oxidative stress as a driving force of ferroptosis may provide new insights into the treatment of neurological disorders^46^.

FTO demethylase has been implicated in ferroptosis regulation in a context-dependent manner. In a Parkinson’s disease model, silencing FTO alleviates ferroptosis in an m6A-Nrf2-dependent manner^47^. Conversely, in oral squamous cell carcinoma, FTO promotes ferroptosis by suppressing ACSL3 and GPX4 expression^48^. These contrasting findings suggest that FTO’s role in ferroptosis is highly cell-type and context-specific. In the present study, pharmacological inhibition of FTO using MO-I-500 attenuated erastin-induced oxidative stress in microglial cells (Fig. 3). Specifically, we found that the addition of MO-I-500 significantly decreased intracellular ROS levels (Fig. 3A–C), mitochondrial superoxide levels (Fig. 3D), and lipid accumulation (Fig. 3E). This is consistent with our previous observations showing antioxidant and cytoprotective effects of MO-I-500 in streptozotocin-treated astrocytes^38^ LPS-treated suprachiasmatic nucleus cells^49^, and TBHP-treated neuroblastoma cells^35^. Together, these findings support a modulatory role for FTO inhibition in oxidative stress-driven cellular responses, including those associated with ferroptosis.

Lipid peroxidation is a critical driver of ferroptosis^50^. The accumulation of lipid peroxides damages the cell membrane, leading to cell death. Iron catalyzes the formation of reactive oxygen species, which initiate lipid peroxidation^51^. GPX4 plays a crucial role in mitigating lipid peroxidation, and its deficiency leads to increased lipid peroxidation and susceptibility to ferroptosis^52^. In oral squamous carcinoma cells (OSCC), high FTO expression is associated with ferroptosis, as FTO suppresses GPX4 expression, thereby promoting lipid peroxidation^48^. In this study, lipid accumulation was assessed using a BODIPY-based flow cytometry approach, revealing that MO-I-500 reduced lipid accumulation in erastin-treated microglial cells (Fig. 3E). While this suggests that FTO inhibition may influence lipid metabolic remodeling associated with ferroptotic stress, the precise molecular mechanisms linking FTO activity to lipid handling remain to be elucidated.

Mitochondrial morphological changes, including swelling, cristae loss, outer membrane rupture, and altered mitochondrial dynamics, are hallmarks of ferroptosis^53^. However, the role of mitochondria in ferroptosis remains controversial. Previous studies show that cells lacking mitochondria can still undergo ferroptosis^54^. Conversely, mitochondria appear to be critical in cysteine-deprivation-induced ferroptosis^55^. In our study, erastin treatment did not significantly alter mitochondrial mass or mitochondrial membrane potential in C8-B4 cells (Fig. 4). These findings indicate that, under the experimental conditions tested, erastin-induced ferroptotic stress occurs without detectable changes in these mitochondrial parameters. However, because mitochondrial respiration, ATP production, and ultrastructural morphology were not assessed, we cannot exclude more subtle or functional mitochondrial alterations. Therefore, our conclusions regarding mitochondrial involvement are limited to mass and membrane potential measurements.

In addition to functional readouts of ferroptotic stress, we assessed the expression of ferroptosis-related proteins, including GPX4, ACSL4, and ferritin heavy chain (FTH1). Western blot analysis revealed no significant changes in GPX4 or ACSL4 protein levels following erastin treatment or erastin + MO-I-500 co-treatment (Fig. S5).

While GPX4 and ACSL4 are canonical regulators of ferroptosis, their expression levels do not necessarily change in all ferroptotic contexts. Genetic network analyses have shown that ACSL4 is not universally required for ferroptosis and that its role can be highly stimulus- and cell-type dependent, even though it contributes to lipid metabolic remodeling associated with susceptibility. Moreover, although GPX4 inhibition of activity is fundamental to ferroptosis biochemistry, ferroptotic cell death can proceed via GPX4-independent mechanisms, and expression changes in GPX4 are not always observed across models. Taken together, the absence of statistically significant changes in GPX4 and ACSL4 expression in C8-B4 microglia under our conditions is consistent with the context-dependent regulation of these markers in ferroptotic signaling^40,56^.

We also examined FTH1 expression as an indicator of iron storage and cellular iron handling. Although trends toward modulation of FTH1 expression were observed, these changes did not reach statistical significance (Fig. S5). This suggests that acute erastin exposure in C8-B4 cells does not markedly alter ferritin expression and that iron-dependent oxidative stress in this model may be driven by changes in labile iron availability or iron utilization rather than bulk ferritin regulation. Together, these data indicate that ferroptotic signaling in this microglial model is regulated predominantly at a functional and metabolic level rather than through robust changes in the expression of canonical ferroptosis-associated proteins.

Ferroptosis has also been linked to dysregulated microglial behaviour^57^. Activated or stressed microglia accumulates iron and exhibit altered migratory properties, which may contribute to neuroinflammation and disease progression^29,58^. In our model, erastin enhanced microglial migration, potentially reflecting a stress-induced motile phenotype. FTO inhibition significantly reduced this erastin-induced migration, suggesting that modulation of oxidative stress and lipid accumulation may indirectly influence microglial motility (Fig. 2). Nevertheless, migration alone does not define microglial activation, and these findings should be interpreted as changes in functional behaviour rather than definitive activation states.

Despite the insights provided by this study, several limitations should be acknowledged. Classical markers of microglial activation, including inflammatory cytokines, receptors, and activation-associated proteins, as well as direct assessment of the Nrf2 signaling pathway, were not examined, which limits mechanistic interpretation of the effects of MO-I-500. These aspects warrant further investigation and will be addressed in future studies to more fully define the molecular mechanisms underlying FTO-dependent regulation of ferroptosis in microglia.

In conclusion, our study demonstrates that pharmacological inhibition of FTO using MO-I-500 attenuates several oxidative and lipid-related features associated with erastin-induced ferroptotic stress in microglial cells. While these findings support a modulatory role for FTO in ferroptosis-associated cellular responses, further studies are required to define the precise molecular mechanisms involved, including direct assessment of m6A-dependent targets and canonical ferroptosis rescue pathways. Future work should also explore the relevance of these findings in more complex in vivo models of neuroinflammation and neurodegeneration.

## Supplementary Information

Below is the link to the electronic supplementary material.

Supplementary Material 1

## Data Availability

The datasets generated during and/or analysed during the current study are available from the corresponding author on reasonable request. Uncropped Western blot images are included in the Supplementary Data.

## References

[1] Hirschhorn, T. & Stockwell, B. R. The development of the concept of ferroptosis. *Free Radical Biology and Medicine* vol. 133 Preprint at (2019). 10.1016/j.freeradbiomed.2018.09.043 10.1016/j.freeradbiomed.2018.09.043 PMC6368883 30268886

[2] Zhou, B. et al. Insights into targeted ferroptosis in mechanisms, biology, and role of Alzheimer’s disease: an update. *Front. Aging Neurosci.* **17**, 1587986 (2025). 40761698 10.3389/fnagi.2025.1587986 PMC12319056

[3] Stockwell, B. R. Ferroptosis turns 10: emerging mechanisms, physiological functions, and therapeutic applications. *Cell* **185**, 2401–2421 (2022). 35803244 10.1016/j.cell.2022.06.003 PMC9273022

[4] Yan, H. et al. Ferroptosis: mechanisms and links with diseases. *Signal Transduction and Targeted Therapy* vol. 6 Preprint at (2021). 10.1038/s41392-020-00428-9 10.1038/s41392-020-00428-9 PMC7858612 33536413

[5] Ru, Q. et al. Iron homeostasis and ferroptosis in human diseases: mechanisms and therapeutic prospects. *Signal. Transduct. Target. Therapy*. **9** (1), 9–271 (2024). (2024). 10.1038/s41392-024-01969-z PMC11486532 39396974

[6] Li, F. J. et al. System Xc–/GSH/GPX4 axis: an important antioxidant system for the ferroptosis in drug-resistant solid tumor therapy. *Front. Pharmacol.* **13**, 910292 (2022). 36105219 10.3389/fphar.2022.910292 PMC9465090

[7] Lewerenz, J. et al. The Cystine/Glutamate Antiporter System xc – in Health and Disease: from molecular mechanisms to novel therapeutic opportunities. *Antioxid. Redox Signal.* **18**, 522 (2013). 22667998 10.1089/ars.2011.4391 PMC3545354

[8] Li, F. J. et al. System Xc–/GSH/GPX4 axis: An important antioxidant system for the ferroptosis in drug-resistant solid tumor therapy. *Front. Pharmacol.*10.3389/fphar.2022.910292 (2022). 10.3389/fphar.2022.910292 PMC9465090 36105219

[9] Jiang, Y., Glandorff, C. & Sun, M. G. S. H. Ferroptosis: side-by-side partners in the fight against tumors. *Antioxidants* **13**, 697 (2024). 38929136 10.3390/antiox13060697 PMC11201279

[10] Costa, I. et al. Molecular mechanisms of ferroptosis and their involvement in brain diseases. *Pharmacol. Ther.* **244**, 108373 (2023). 36894028 10.1016/j.pharmthera.2023.108373

[11] Li, J. et al. Ferroptosis: past, present and future. *Cell Death Dis.* **11** (2), 11–88 (2020). (2020). 32015325 10.1038/s41419-020-2298-2 PMC6997353

[12] Zhou, Q. et al. Ferroptosis in cancer: from molecular mechanisms to therapeutic strategies. *Signal Transduction Targeted Ther.*10.1038/s41392-024-01769-5 (2024). 10.1038/s41392-024-01769-5 PMC10920854 38453898

[13] Xu, Y. et al. The role of ferroptosis in neurodegenerative diseases. *Mol. Biol. Reports*. 10.1007/s11033-022-08048-y (2023). 10.1007/s11033-022-08048-y 36385663

[14] Chen, Y. et al. Ferroptosis: a novel therapeutic target for ischemia-reperfusion injury. *Front. Cell Dev. Biol.*10.3389/fcell.2021.688605 (2021). 10.3389/fcell.2021.688605 PMC8384466 34447746

[15] Ma, H., Dong, Y., Chu, Y., Guo, Y. & Li, L. The mechanisms of ferroptosis and its role in alzheimer’s disease. *Front. Mol. Biosci.*10.3389/fmolb.2022.965064 (2022). 10.3389/fmolb.2022.965064 PMC9459389 36090039

[16] Lin, K. J. et al. Iron Brain Menace: The Involvement of Ferroptosis in Parkinson Disease. *Cells*. 10.3390/cells11233829 (2022). 10.3390/cells11233829 PMC9735800 36497089

[17] Wang, T. et al. Ferroptosis mediates selective motor neuron death in amyotrophic lateral sclerosis. *Cell Death Differ* **29**, 1187–1198 (2022). 10.1038/s41418-021-00910-z PMC9177596 34857917

[18] Hanslik, K. L., Marino, K. M. & Ulland, T. K. Modulation of Glial function in health, aging, and neurodegenerative disease. *Front. Cell. Neurosci.* **15**, 718324 (2021). 34531726 10.3389/fncel.2021.718324 PMC8439422

[19] Garland, E. F., Hartnell, I. J. & Boche, D. Microglia and astrocyte function and communication: what do we know in humans? *Front. Neurosci.* **16**, 824888 (2022). 35250459 10.3389/fnins.2022.824888 PMC8888691

[20] Purushotham, S. S. & Buskila, Y. Astrocytic modulation of neuronal signalling. *Front. Netw. Physiol.* **3**, 1205544 (2023). 37332623 10.3389/fnetp.2023.1205544 PMC10269688

[21] Pearson-Leary, J., Osborne, D. M. & McNay, E. C. Role of Glia in stress-induced enhancement and impairment of memory. *Front Integr. Neurosci.* **9**, (2016). 10.3389/fnint.2015.00063 PMC4707238 26793072

[22] Camberos-Barraza, J. et al. Sleep, Glial Function, and the Endocannabinoid System: Implications for Neuroinflammation and Sleep Disorders. *Int J. Mol. Sci* **25**, (2024). 10.3390/ijms25063160 PMC10970053 38542134

[23] Yang, G. et al. Microglia-orchestrated neuroinflammation and synaptic remodeling: roles of pro-inflammatory cytokines and receptors in neurodegeneration. *Front. Cell. Neurosci.* **19**, 1700692 (2025). 41292555 10.3389/fncel.2025.1700692 PMC12641016

[24] Rábago-Monzón, Á. R. et al. Stress-induced sleep dysregulation: the roles of astrocytes and microglia in neurodegenerative and psychiatric disorders. *Biomedicines* **13**, 1121 (2025). 40426947 10.3390/biomedicines13051121 PMC12109018

[25] Zhou, X. et al. Mitophagy and cGAS–STING crosstalk in neuroinflammation. *Acta Pharm. Sin B*. **14**, 3327 (2024). 39220869 10.1016/j.apsb.2024.05.012 PMC11365416

[26] Allen, N. J. & Lyons, D. A. Glia as architects of central nervous system formation and function. *Science* **362**, 181–185 (2018). 30309945 10.1126/science.aat0473 PMC6292669

[27] Hristovska, I. et al. Sleep decreases neuronal activity control of microglial dynamics in mice. *Nat. Commun.* **13**, (2022). 10.1038/s41467-022-34035-9 PMC9586953 36271013

[28] Ryan, S. K. et al. Microglia ferroptosis is regulated by SEC24B and contributes to neurodegeneration. *Nat. Neurosci.* **26**, 12–26 (2023). 10.1038/s41593-022-01221-3 PMC9829540 36536241

[29] Liu, S., Gao, X. & Zhou, S. New target for prevention and treatment of neuroinflammation: microglia iron accumulation and ferroptosis. *ASN Neuro.*10.1177/17590914221133236 (2022). 10.1177/17590914221133236 PMC9607999 36285433

[30] Fan, B. et al. Summary of the mechanism of ferroptosis regulated by m6A modification in cancer progression. *Front. Cell. Dev. Biol.* **13**, 1507171 (2025). 40271153 10.3389/fcell.2025.1507171 PMC12014555

[31] Zhang, J., Qiu, T., Yao, X. & Sun, X. Insights into the role of N6-methyladenosine in ferroptosis. *Biomed. Pharmacother.*10.1016/j.biopha.2023.115192 (2023). 10.1016/j.biopha.2023.115192 37487443

[32] Li, L., Zeng, J., He, S., Yang, Y. & Wang, C. METTL14 decreases FTH1 mRNA stability via m6A methylation to promote sorafenib-induced ferroptosis of cervical cancer. 10.1080/15384047.2024.2349429 (2024). 10.1080/15384047.2024.2349429 PMC11093024 38738555

[33] Li, X., Yang, G., Ma, L., Tang, B. & Tao, T. N6-methyladenosine (m6A) writer METTL5 represses the ferroptosis and antitumor immunity of gastric cancer. *Cell Death Discovery.* **10**, 1–12 (2024). 10.1038/s41420-024-02166-1 PMC11390903 39261486

[34] Zheng, G. et al. Synthesis of a FTO inhibitor with anticonvulsant activity. *ACS Chem. Neurosci.* **5**, 658–665 (2014). 24834807 10.1021/cn500042t PMC4140589

[35] Greco, D. et al. Small molecule FTO inhibitor MO-I-500 protects differentiated SH-SY5Y neuronal cells from oxidative stress. *Front. Mol. Neurosci.* **18**, 1736173 (2026). 41602161 10.3389/fnmol.2025.1736173 PMC12832913

[36] Mohan, S. et al. Role of ferroptosis pathways in neuroinflammation and neurological disorders: from pathogenesis to treatment. *Heliyon* **10**, e24786 (2024). 38314277 10.1016/j.heliyon.2024.e24786 PMC10837572

[37] Liddell, J. R. et al. Microglial ferroptotic stress causes non-cell autonomous neuronal death. *Mol. Neurodegener.* **19**, (2024). 10.1186/s13024-023-00691-8 PMC10840184 38317225

[38] Cockova, Z., Honc, O., Telensky, P., Olsen, M. J. & Novotny, J. Streptozotocin-induced astrocyte mitochondrial dysfunction is ameliorated by FTO inhibitor MO-I-500. *ACS Chem. Neurosci.* **12**, 3818–3828 (2021). 34491720 10.1021/acschemneuro.1c00063

[39] Latunde-Dada, G. O. Ferroptosis: role of lipid peroxidation, iron and ferritinophagy. *Biochim. et Biophys. Acta (BBA) - Gen. Subj.* **1861**, 1893–1900 (2017). 10.1016/j.bbagen.2017.05.019 28552631

[40] Tang, D., Chen, X., Kang, R. & Kroemer, G. Ferroptosis: molecular mechanisms and health implications. *Cell. Res. 2020*. **31:2** (31), 107–125 (2020). 10.1038/s41422-020-00441-1 PMC8026611 33268902

[41] Ren, J. X., Sun, X., Yan, X. L., Guo, Z. N. & Yang, Y. Ferroptosis in neurological diseases. *Front. Cell. Neurosci.* **14**, 554464 (2020). 10.3389/fncel.2020.00218 PMC7370841 32754017

[42] Zhou, Y. et al. Ferroptosis and its potential role in the nervous system diseases. *J. Inflamm. Res.* **15**, 1555 (2022). 35264867 10.2147/JIR.S351799 PMC8901225

[43] Park, M. W. et al. NOX4 promotes ferroptosis of astrocytes by oxidative stress-induced lipid peroxidation via the impairment of mitochondrial metabolism in Alzheimer’s diseases. *Redox Biol.* **41**, (2021). 10.1016/j.redox.2021.101947 PMC8027773 33774476

[44] Tang, Z. et al. NRF2 deficiency promotes ferroptosis of astrocytes mediated by oxidative stress in Alzheimer’s disease. *Mol. Neurobiol.* **61**, 7517–7533 (2024). 10.1007/s12035-024-04023-9 38401046

[45] Yong, Y. et al. A novel ferroptosis inhibitor, Thonningianin A, improves Alzheimer’s disease by activating GPX4. *Theranostics* **14**, 6161–6184 (2024). 39431016 10.7150/thno.98172 PMC11488096

[46] Li, J. et al. Targeting molecular mediators of ferroptosis and oxidative stress for neurological disorders. *Oxid. Med. Cell. Longev.* (2022). 10.1155/2022/3999083 PMC9337979 35910843

[47] Pang, P., Zhang, S., Fan, X. & Zhang, S. Knockdown of fat mass and obesity alleviates the ferroptosis in Parkinson’s disease through m6A-NRF2-dependent manner. *Cell. Biol. Int.* **48**, 431–439 (2024). 38180302 10.1002/cbin.12118

[48] Wang, Z. et al. FTO sensitizes oral squamous cell carcinoma to ferroptosis via suppressing ACSL3 and GPX4. *Int. J. Mol. Sci.* **24**, 16339 (2023). 38003537 10.3390/ijms242216339 PMC10671523

[49] Filipovská, E. et al. The role of N6-methyladenosine RNA methylation in the crosstalk of circadian clock and neuroinflammation in rodent suprachiasmatic nuclei. *Eur. J. Neurosci.* **60**, 4586–4596 (2024). 39007275 10.1111/ejn.16471

[50] Endale, H. T., Tesfaye, W. & Mengstie, T. A. ROS induced lipid peroxidation and their role in ferroptosis. *Front. Cell. Dev. Biol.* **11**, 1226044 (2023). 37601095 10.3389/fcell.2023.1226044 PMC10434548

[51] Rochette, L. et al. Lipid peroxidation and iron metabolism: two corner stones in the homeostasis control of ferroptosis. *Int. J. Mol. Sci.* **24**, (2022). 10.3390/ijms24010449 PMC9820499 36613888

[52] Zhang, W., Liu, Y., Liao, Y., Zhu, C. & Zou, Z. GPX4, ferroptosis, and diseases. *Biomed. Pharmacother.* **174**, 116512 (2024). 38574617 10.1016/j.biopha.2024.116512

[53] Guo, J. et al. Mitochondria as multifaceted regulators of ferroptosis. *Life metabolism*. **1**, 134–148 (2022). 39872359 10.1093/lifemeta/loac035 PMC11749789

[54] Dixon, S. J. et al. Ferroptosis: an iron-dependent form of nonapoptotic cell death. *Cell* **149**, 1060–1072 (2012). 22632970 10.1016/j.cell.2012.03.042 PMC3367386

[55] Gao, M. et al. Role of Mitochondria in Ferroptosis. *Mol. Cell.* **73**, 354–363e3 (2019). 30581146 10.1016/j.molcel.2018.10.042 PMC6338496

[56] Magtanong, L. et al. Context-dependent regulation of ferroptosis sensitivity. *Cell. Chem. Biol.* **29**, 1409–1418e6 (2022). 35809566 10.1016/j.chembiol.2022.06.004 PMC9481678

[57] Wang, M. et al. Revisiting the intersection of microglial activation and neuroinflammation in Alzheimer’s disease from the perspective of ferroptosis. *Chem. Biol. Interact.* **375**, (2023). 10.1016/j.cbi.2023.110387 36758888

[58] Ana, B. Aged-related changes in microglia and neurodegenerative diseases: exploring the connection. *Biomedicines*. **12**, 1737 (2024). 39200202 10.3390/biomedicines12081737 PMC11351943

