# Supplementary material for: Small-molecule FTO inhibitor MO-I-500 protects C8-B4 microglial cells from erastin-induced ferroptosis
Source: Sci Rep. 2026 Apr 18;16:18062. doi: 10.1038/s41598-026-47881-0 (PMC13253875; doi:10.1038/s41598-026-47881-0)
Supplement: Supplementary file 1 — Supplementary Material 1 [file 41598_2026_47881_MOESM1_ESM.docx]

## **Supplementary materials**


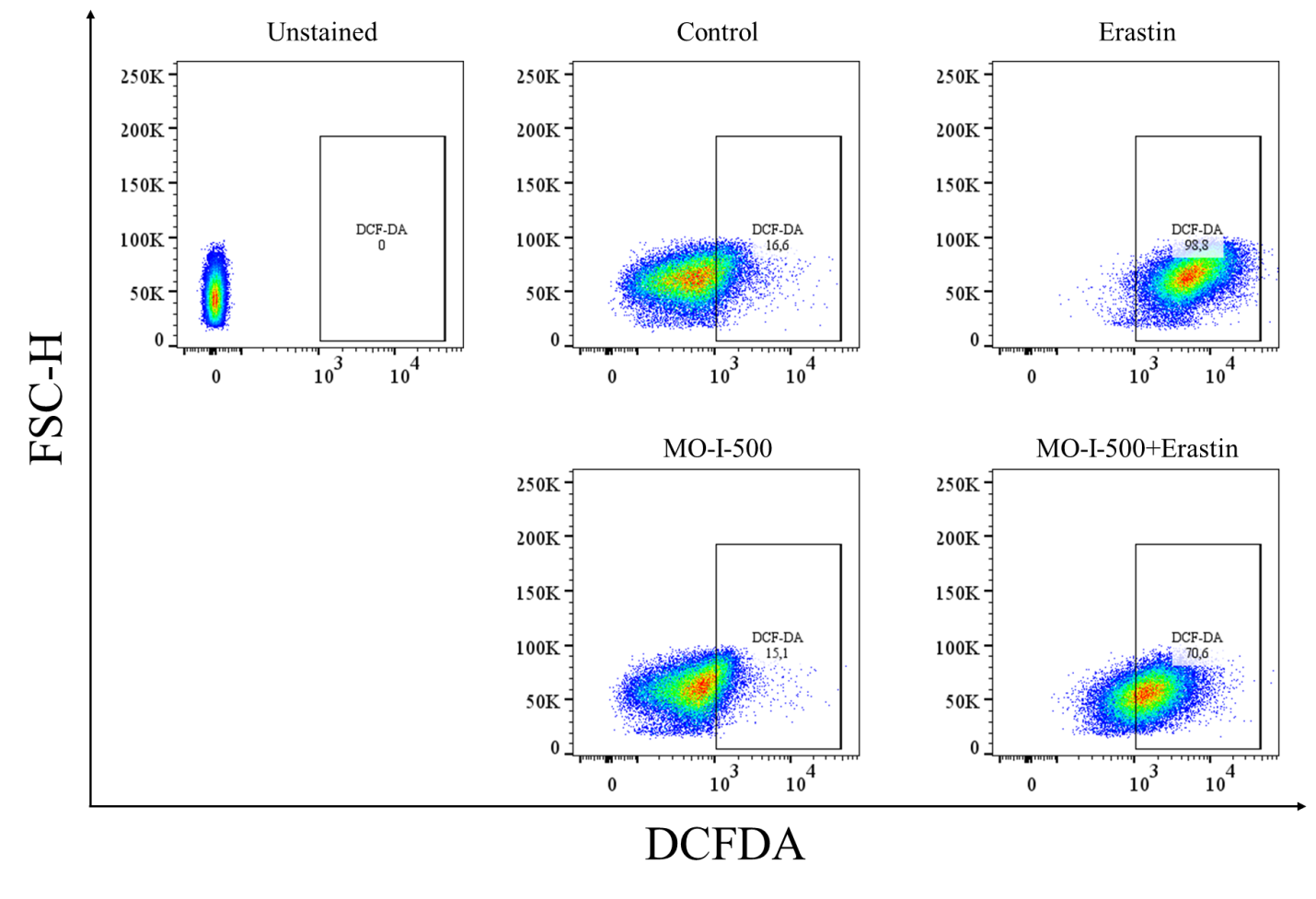


**Supplementary Figure S1. Representative flow cytometry dot plots for intracellular ROS (DCFDA) in C8-B4 cells**. Representative flow cytometry dot plots illustrating DCFDA fluorescence in C8-B4 cells treated with vehicle, erastin, MO-I-500, or erastin + MO-I-500. Gating strategy and positive populations are shown for each treatment. The data shown corresponds to one representative experiment. Quantitative summary of DCFDA fluorescence is presented in Figure 3C of the main manuscript.

##
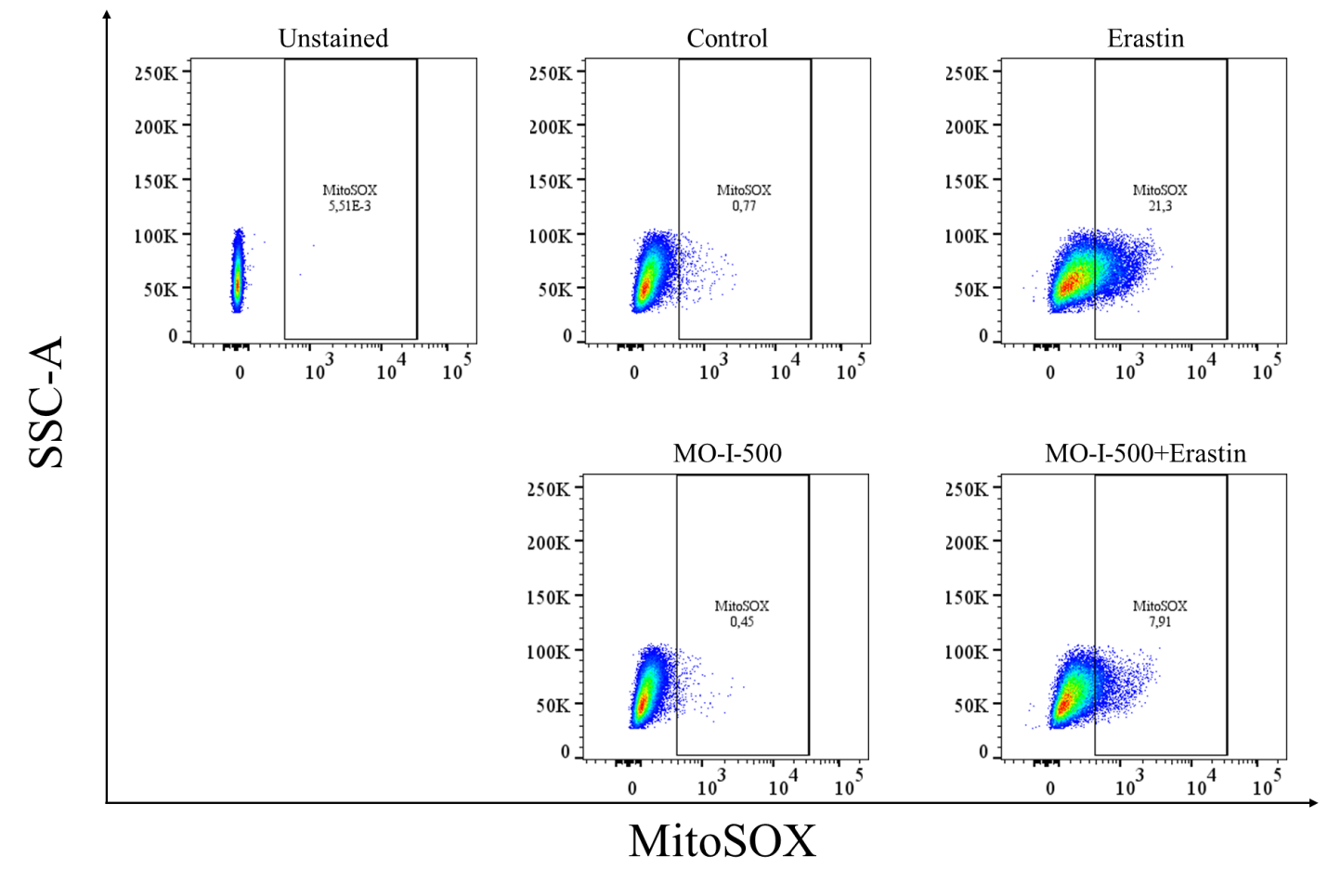


**Supplementary Figure S2. Representative flow cytometry dot plots for mitochondrial superoxide (MitoSOX™ Red) in C8-B4 cells.** Representative flow cytometry dot plots showing MitoSOX™ Red fluorescence in C8-B4 cells treated with vehicle, erastin, MO-I-500, or erastin + MO-I-500. Gating strategies and positive populations are indicated. One representative experiment is shown. Quantitative analysis is provided in Figure 3D of the main manuscript.


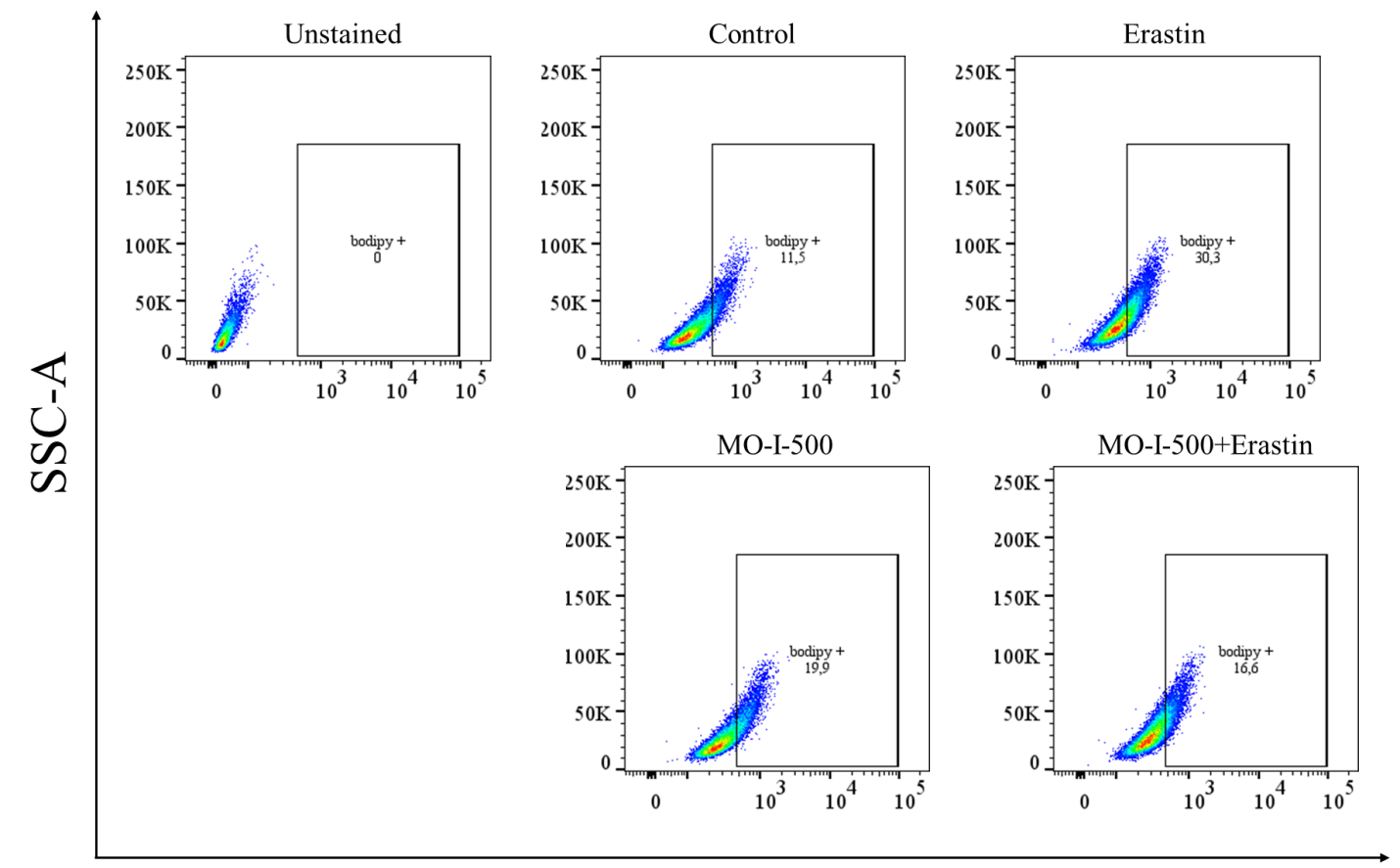


**Supplementary Figure S3. Representative flow cytometry dot plots for BODIPY probe in C8-B4 cells.** Representative flow cytometry dot plots showing BODIPY fluorescence in C8-B4 cells treated with vehicle, erastin, MO-I-500, or erastin + MO-I-500. Gating strategies and positive populations are indicated. One representative experiment is shown. Quantitative analysis is provided in Figure 3E of the main manuscript.


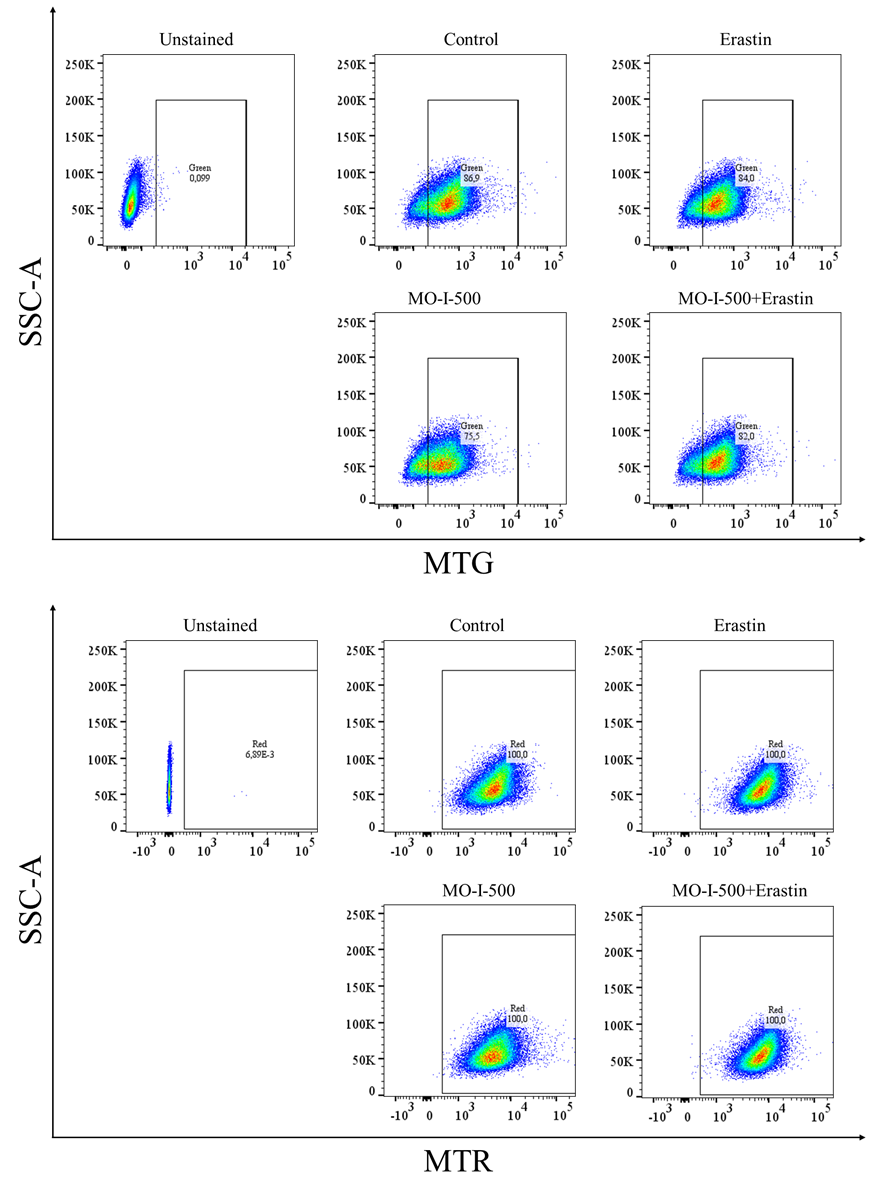


**Supplementary Figure S4. Representative flow cytometry dot plots for mitochondrial mass and membrane potential (MitoTracker™ Green FM and MitoTracker™ Red CMXRos) in C8-B4 cells.** Representative dot plots displaying MitoTracker™ Green FM (mitochondrial mass) and MitoTracker™ Red CMXRos (mitochondrial membrane potential) fluorescence in C8-B4 cells under vehicle, erastin, MO-I-500, or erastin + MO-I-500 treatments. Gating strategy and positive populations are shown. One representative experiment is displayed. Quantitative results are presented in Figure 4 of the main manuscript.

**Expression of canonical ferroptosis and iron-responsive markers.**

To further characterize ferroptotic signaling, we examined the expression of canonical ferroptosis markers (GPX4 and ACSL4) and the iron storage protein FTH1 via Western blot. Neither GPX4 nor ACSL4 expression was significantly altered following erastin treatment or co-treatment with MO-I-500. Similarly, FTH1 expression did not show statistically significant changes across treatment conditions (Supplementary Fig. S5). These results indicate that erastin-induced ferroptosis in C8-B4 microglia occurs independently of transcriptional or translational regulation of canonical ferroptosis proteins or ferritin-mediated iron sequestration.


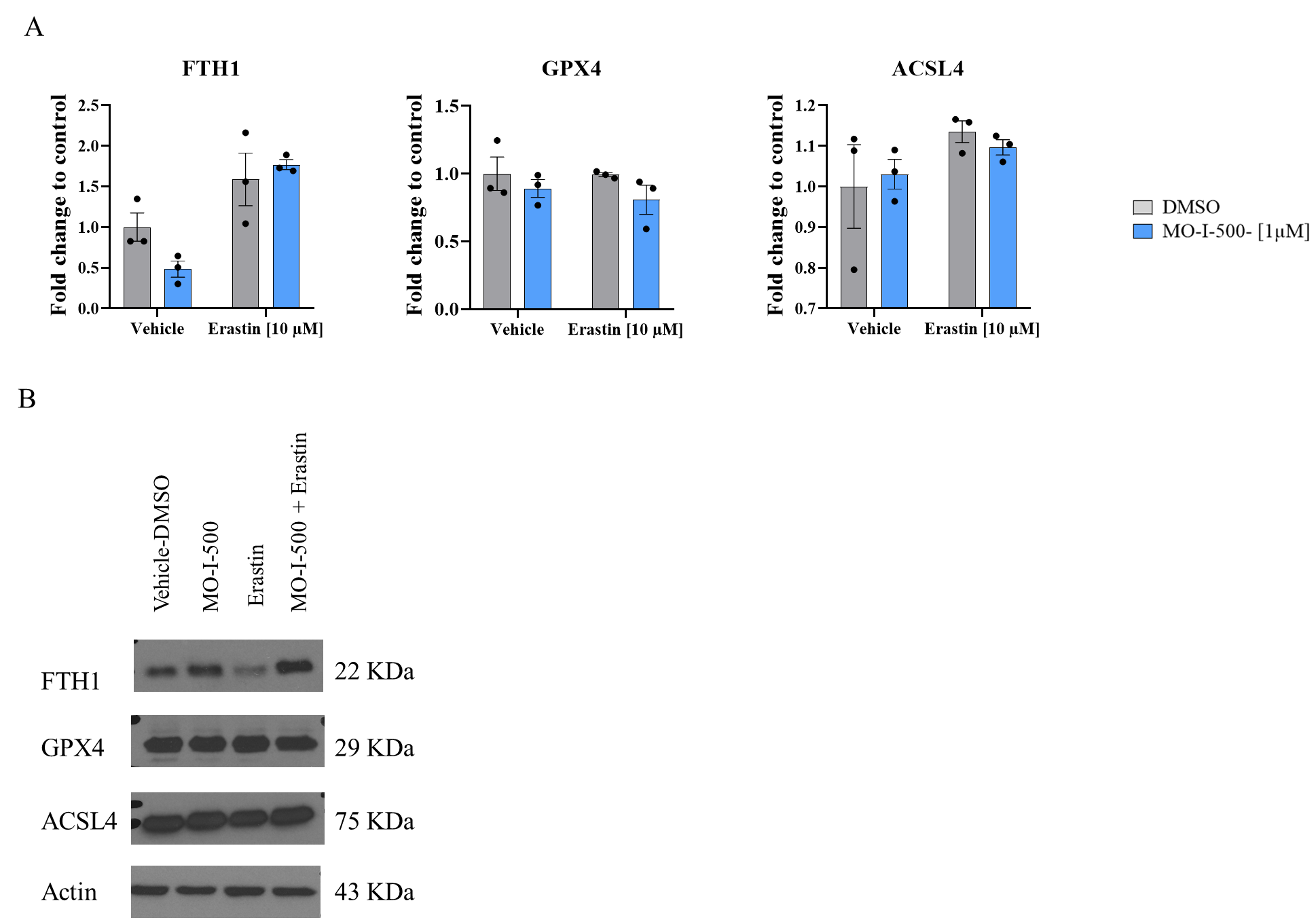


**Supplementary Figure S5. Expression of ferroptosis markers**. Western blot analysis of GPX4, ACSL4, and FTH1 protein expression in C8-B4 cells treated with erastin and/or MO-I-500. Representative blots and quantification, normalized to β-actin, are shown (A-B). Data are expressed as mean ± SEM from n = 3 independent experiments. No statistically significant differences were observed.


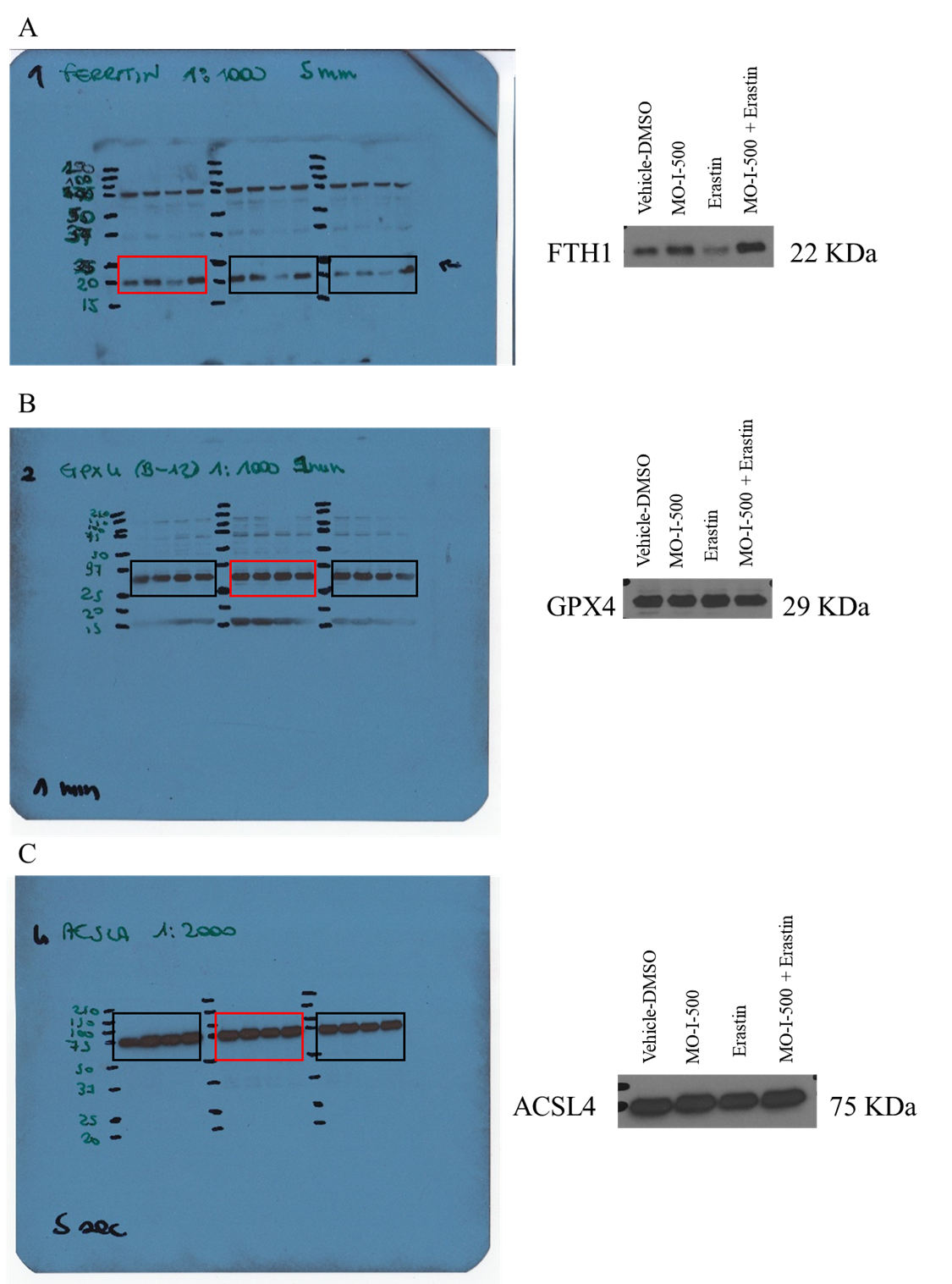


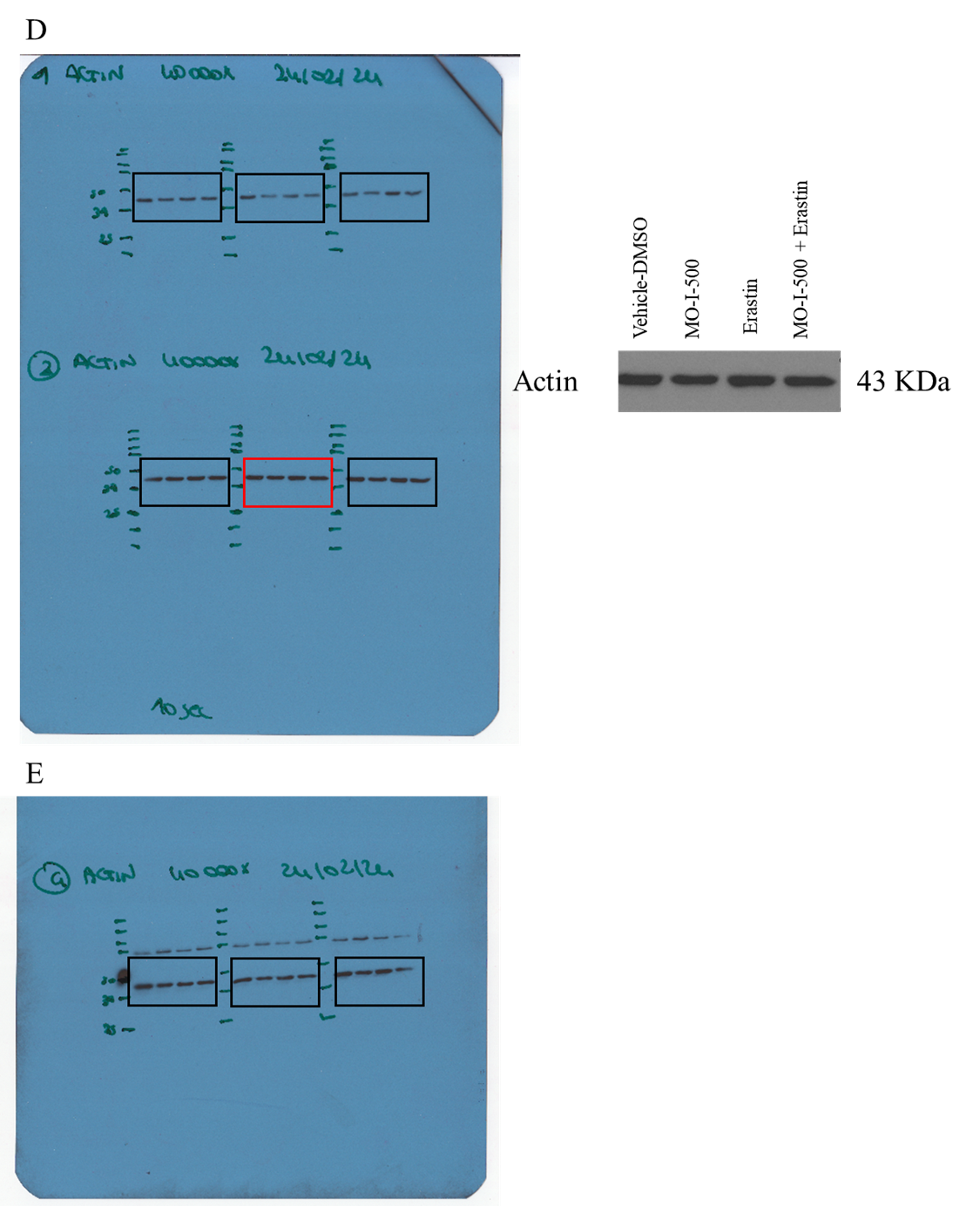


**Supplementary Figure S6. Expression of ferroptosis markers. Uncropped images of Western blot analysis.**

Uncropped Western blot images corresponding to the results presented in Supplementary Figure S5 are shown for FTH1 (A), GPX4 (B), ACSL4 (C), and β-actin (D–E). The samples displayed as representative images in Supplementary Figure S5B are indicated by red boxes. Red and black boxes denote the three sets of samples used for each experiment.
